# Understanding genome structural variations

**DOI:** 10.18632/oncotarget.6485

**Published:** 2015-12-07

**Authors:** Alexej Abyzov, Shantao Li, Mark B. Gerstein

**Affiliations:** Department of Health Sciences Research, Center for Individualized Medicine, Mayo Clinic, Rochester, Minnesota, USA

**Keywords:** Chromosome Section, genome, structural, variation, breakpoint

Genome structural variations (SVs) in the human genome are defined as DNA sequence polymorphisms of at least a few dozen or few hundred bases in length and include deletions, duplications, inversions, translocation, retroelement insertions, and more complex rearrangements that could be thought of as consisting of multiple fragments from the just listed categories. More bases in a personal genome are affected by SVs than by single nucleotide polymorphisms (SNPs), suggesting that SV have a larger or comparable effect on personal phenotype than SNPs. SVs frequently occur in tumor genomes, with several tumor types (e.g., ovarian) having SVs as the dominant type of genomic alteration. Numerous *de novo* SVs have been linked to various diseases.

Because of their size and enrichment in repeat regions, these are the most challenging variants to discover and analyze. Even more challenging is the precise identification of SV breakpoints at a single base pair resolution. But reward is huge, as precise breakpoints hold invaluable information about the origin of each SV; i.e., about the mutational process that created it. The main mechanisms of SV mutagenesis are largely known or hypothesized based on existing evidence [1]: Non-Allele Homologous Recombination (NAHR), Non-Homologous End Joining (NHEJ), Microhomology-Mediated End Joining (MMEJ), errors during replication (replicative mechanisms), and retroelement insertions. However, the details of how they generate SVs are still to be uncovered.

The 1,000 Genomes Project, specifically aimed at the analysis of genomic variants across 2,504 individuals from 26 diverse human sub-populations, provided one of the largest data resources to date. Analysis of the data allowed precise reconstruction of breakpoints for over 30 thousand germline SVs, while, in turn, analyses of their breakpoints revealed details of mutation mechanisms generating SVs [2-4].

The classical NAHR mechanism postulates meiotic cell division as a requirement for generating a germline SV, which happens during chromosomal crossover. Interestingly, breakpoints with a signature of NAHR (i.e., with long sequence homologies) found by the project were associated with open chromatin, higher DNA accessibility, and active histone marks in mitotically dividing cells [2]. Such associations were specific to NAHR breakpoints and could not be fully explained by recombination rate, segmental duplication, or repeat content. Therefore, besides recombination during meiosis there could be other circumstances when such SVs are generated. In particular, these associations imply that NAHR-like mutagenesis can happen in non-dividing cells during the repair of double stranded DNA breaks [2], and thus support the proposed intramolecular NAHR. It was also noted that such mutagenesis could also explain extrachromosomal circular DNA (eccDNA), while distribution of eccDNA origins across genome was consistent with the mutagenesis [2].

Breakpoints of SVs generated by NHEJ and replicative mechanisms were known to often be non-blunt; i.e., to include a few extra bases at sequence junctions and sometimes being rather complex. Analysis of 1,651 complex deletions, which are thought to be exclusively created by replicative mechanisms, allowed classifying patterns of rearrangements around breakpoints into few but inclusive classes [3]: deletion with insertion, deletion with insertion and duplication, deletion with insertion and multiple duplications, multiple deletions separated by a forward or inverted spacer, and deletion with inversion. Analysis of origin for the duplicated sequence revealed two characteristic locations relative to breakpoints - 20 to 60 bps and 2 to 6 kbps - and their generally later replication than the locations of breakpoints [2]. While these observations are likely to be related to the way SVs are generated during replication (e.g., they may suggest the coiling of DNA around the replication bubble), their exact meaning is yet to be deciphered.

Mechanism for insertions of retrotransposable elements through reverse transcription of their mRNA is well characterized. Still, large-scale analysis revealed peculiar preference of transposon integration complexes for hypomethylated DNA [2]. Additionally, analysis of rare cases - when reverse transcriptase mistakenly integrates the mRNA of regular genes, thereby creating a processed pseudogene - exposed the association of gene expression during a cycle with pseudogene generation rate [4]. The closer maximum gene expression was to the end of metaphase the more pseudogenes it had on average, suggesting the coupling of retrotransposition to cell division [4].
